# Acorn Isotopic Composition: A New Promising Tool for Authenticity Maps of Montado’s High-Value Food Products

**DOI:** 10.3390/molecules25071535

**Published:** 2020-03-27

**Authors:** Carla Alegria, Cristina Antunes, Manuela Giovanetti, Marta Abreu, Cristina Máguas

**Affiliations:** 1 cE3c—Centre for Ecology, Evolution and Environmental Changes, Faculdade de Ciências, Universidade de Lisboa, Campo Grande, 1749-016 Lisboa, Portugal; cmaantunes@fc.ul.pt (C.A.); manuela.giovanetti@gmail.com (M.G.); 2 Unidade Tecnologia e Inovação, Instituto Nacional de Investigação Agrária e Veterinária, I.P. Av. da República, Quinta do Marquês, 2780-157 Oeiras, Portugal; marta.abreu@iniav.pt; 3 CREA—Research Centre for Agriculture and Environment, Via di Saliceto 80, 40128 Bologna, Italy; 4 LEAF—Linking Landscape, Environment, Agriculture and Food, Instituto Superior de Agronomia, Universidade de Lisboa, Tapada da Ajuda, 1349-017 Lisboa, Portugal

**Keywords:** *Quercus rotundifolia*, *Quercus suber*, physiological traits, δ^15^N, aridity index, isoscapes

## Abstract

It is often overlooked that even food production is linked to the ecology of plants and animals. Living organisms respond to environmental short-and long-term variability: acknowledging this may help in the ultimate goal of valorizing a territory/product. We investigated acorns of the two main *Quercus* species of the Portuguese *Montado*, a main feed of the renown black Iberian pig. We tested their responses to an aridity gradient by morphological parameters and isotopic signature. *Q. rotundifolia* and *Q. suber* acorns did not differ morphologically, even if a higher variability in all parameters was observed in acorns of *Q. suber*. According to the site-specific Aridity Index, correlations are indicative to higher weight and length only in *Q. suber* acorns from more arid sites. As for isotopic composition, there were no differences in nitrogen or carbon (δ^15^N and δ^13^C) between the two species. However, combining the samples and testing for association with the Aridity Index, we found that more arid sites lead to a ^15^N enrichment. This result, combined with the positive correlation between AI and acorns length, support the use of acorns as a tool, their isoscapes of nitrogen being a stepping stone for the provenance of the black Iberian pig.

## 1. Introduction

Consumer demand for food quality is growing along with concerns related to geographic origin, health, and sustainable production. Thus, food products have to respond to current ethical, environmental, and socially sustainable claims. Within the Portuguese Montado landscape, a high nature value farmland, several unique traditional food products of an exceptional quality play a fundamental role in local socioeconomic activities and in the conservation of cultural and natural heritage [1]. The Montado agro-forestry system is a diverse habitat, with different acorn producing oaks (*Quercus suber* L. and *Q. rotundifolia* Lam., 20–80 trees/ha). Nowadays, yields range from 726 to 2450 × 10^3^ t/year [2], and 41% is devoted to feed the Alentejano pig breed (Portuguese registration for the black Iberian pig), and wild fauna. A consistent amount, around 55% of acorn production, is left on the fields [3] so far unvalued.

The *Montado* agrosilvopastoral ecosystem is a multi-layered system that combines forest harvesting, extensive livestock husbandry, pastures and/or cereal cultivation, and other traditional uses (hunting, beekeeping, and mushroom picking). Livestock production is a dominant activity and one of the most important sources of income to rural communities [4,5]. Among the domestic animals, well-adapted autochthonous breeds predominate such as the Alentejano pig breed. Its meat has exceptional organoleptic and metabolic characteristics, imprinting to the derived products an extraordinary flavor and exquisite aroma. Most of the resulting products are certified under a Protected Designation of Origin (PDO) or Protected Geographical Indication (PGI), with a very high economic value. 

The differentiation results from the dietary scheme that characterizes the extensive livestock production practiced in *Montado*. The Alentejano pig is allotted to graze during the *montanheira* (pannage) period from October to February, precisely linked with the acorn production. During this period, pigs are entirely dependent on natural resources (acorns and grasses) since legal requirements of the high-value Iberian pig meat and cured products does not allow any supplementary feed during this period [6]. Thus, the sustainability of this extensive livestock husbandry is related to the prevalence of pastures and, especially, acorn production.

Acorn chemical composition varies depending on *Quercus* species, production systems, and climatic and geographic variables, which ultimately affect its nutritive value. In general, acorn composition is comprised mainly of starch (~50%) and 2–5% protein (gluten free) [7]. Acorn fat content is species dependent, ranging from 2% to 30% [7,8], with oleic, palmitic, and linoleic acids as major fatty acids found in acorns. Acorn chemical composition is also strongly influenced by climatic variables (precipitation and temperature) [9], physiological characteristics [10], and genetic and local environmental variability [10]. Regarding productivity, managed *Montado* systems have a 10 times higher production yield of acorns compared to unmanaged *Quercus* forests [11]. This fact highlights the need to evaluate the effects of environmental and human influence on acorn production, considering geographical, climatic, soil, and land use land cover variables.

Since variability in acorn quality may also directly influence products linked to it, clear geographical references can increase food products competitiveness and even broaden the range of consumers based on products reputation. When linked to the uniqueness of the environment, the geographical origin can be verified and traced. Among methodological approaches to discriminate geographical origin, stable isotope ratio analysis is considered the most reliable tool for fingerprinting [12,13,14,15,16,17]. Isotope ratio mass spectrometry (IRMS) allows to differentiate compounds from a unique isotopic signature, imprinted by biochemical and physiological processes, climate, geography, geology, agricultural practices, and processing factors [12,13,18]. The integration of isotopic data with GIS and statistical models enables the development of isoscapes. Isoscapes can be described as “maps” translating the complex spatiotemporal information gathered from IRMS to an interpolated isotope landscape [18].

Several studies regarding the use of stable isotope ratio analysis to discriminate the Iberian pig feeding regime and even pig breeds are known [19,20,21,22,23]. However, focus is on the analysis of the fatty acid isotopic composition (by Gas Chromatography-Combustion-Isotope Ratio Mass Spectrometry, GC-C-IRMS) particularly comparing the ^13^C/^12^C ratio of subcutaneous adipose tissue lipids, as it is most influenced by the animals’ diet. Notwithstanding, these studies [19,20,21,22,23] clearly demonstrate the possibility of distinguishing between Iberian pigs raised traditionally (only on acorns and grass during the “montanheira” period) and animals fed with other food resources (most commonly a mixture of maize, barley, and soy, possibly with added fats) based on the variabilities of δ^13^C of plant products. In this context, we hypothesize that if, we were able to discriminate acorn origin (both geographic and species) based on the isotopic signatures of acorn bulk samples (simplifying the sample preparation procedure) and streamlining results with the development of isoscapes, it would provide information regarding both Iberian pig products provenance (geographical origin) and agricultural production system status.

This preliminary study evaluated the potential use of acorns as authenticity tracers of the Alentejano pig using IRMS analysis of acorn bulk samples. This evaluation considered isotopic responses of acorns from the two main *Quercus* species along a climatic gradient (aridity). From it, we produced easy-reading acorns isoscapes to implement the description of the geographical origin of food products derived from the black Iberian pig. Moreover, this information will also add an extra-value to a future employment of remaining acorns for other dietary products.

## 2. Results and Discussion

Acorns are morphologically classified as a one-seed nut, absent of endosperm and with an achlorophyllous embryo. Even though it is common to use morphological classification to group acorns, phylogenetic and ecological factors are responsible for differences among acorns of *Quercus* species. Efforts have been developed to correlate morphologic characteristics such as shape and size with ecological factors (e.g., edapho-climatic variables, water availability, and vegetation type) [10,24]. However, there is great variability in seed size within and between oak species [10,25] and it has been shown that acorn size is positively correlated with the length of its development period and with rainfall [26].

Our results considered all the most common morphological traits (Table 1), of the two *Quercus* species (*Q. rotundifolia* (QR) and *Q. suber* (QS)). Results indicate no significant differences (*p* > 0.05) regarding acorns’ weight (5.64 and 6.18 g for QR and QS, respectively), length (37.57 and 33.80 mm for QR and QS, respectively), diameter (14.75 and 15.49 mm for QR and QS, respectively), or volume (4.34 and 4.51 cm^3^ for QR and QS, respectively). These results are in agreement with the variability observed in previous literature [10,25].

QS samples denoted the highest variability (Table 1, minimum and maximum values), with broader ranges than those found in QR samples. Variability is likely to be linked with individuals’ genetics, climate, soil, and stand structure [10]. Carborenro et al. [27] reported high variability regarding acorn size and weight between holm-oak individuals. Reported holm oak weight ranges from 1.2 to 6.5 g [10], lower than the ones determined in QR samples. As for QS acorns weight, mean values of 7.69 g have been reported [28], which also agrees with our results.

Relationships between acorn weight and climate exist: higher values were determined in non-continental sites in comparison to continental [29]. Within species, our data support such relation by the correlations found with the site-specific Aridity Index (AI), as shown in Table 2. The AI is a key parameter in drought characterization, extensively used for the evaluation of aridity trends, particularly in the Mediterranean region [30,31]. Generally, lower AI values imply more arid/drier sites, where the climate tends to drought and water resources scarcity. In opposition, higher AI values are usually associated with more humid areas.

As it can be observed in Table 2, AI is only negatively correlated with weight and length of QS samples. These correlations are indicative of higher weight and length in QS acorns from more arid sites (lower AI), suggesting a reduction in QS acorns size in response to AI decrease (humid sites). Therefore, it can be inferred that *Q. suber* investment in seeds could be influenced by plants’ adaptation to water stress and environmental variables, resulting in a survival strategy adequate to the environmental conditions. On the other hand, the morphological traits of QR samples showed no significant correlations with AI. The lack of significance could be justified by the narrower AI range between QR sample collection sites (0.422–0.582 for QR vs. 0.446–0.627 for QS). In fact, as can be observed in Materials and Methods section, all QR collection sites, except one, are classified as semi-arid. Nonetheless, the negative trends (*p* > 0.05) between QR acorn morphological traits and AI hint a similar behavior to the one found for QS acorns.

**Figure 1:**
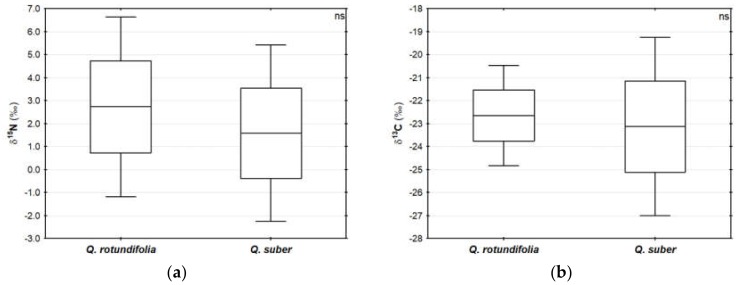

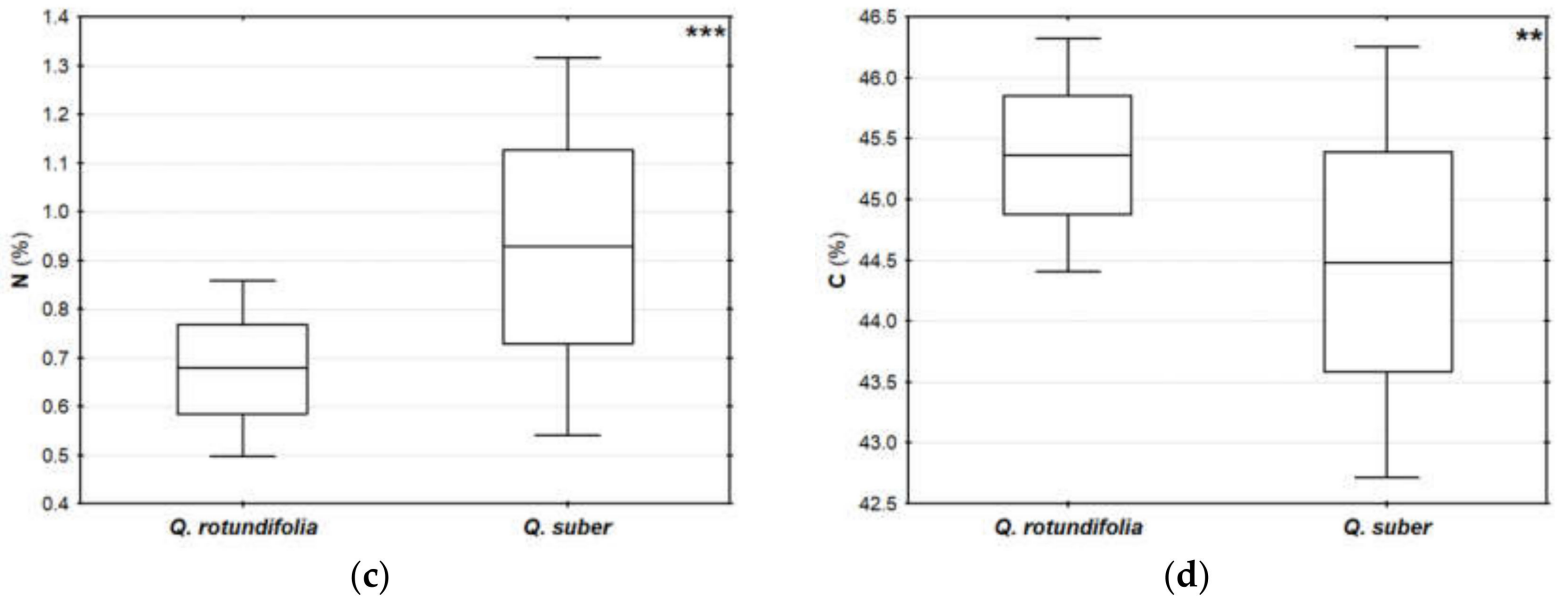
Physiological traits of acorns in two *Quercus* species (*Q. rotundifolia* (n = 15) and *Q. suber* (n = 13)): (**a**) δ^15^N; (**b**) δ^13^C; (**c**) Nitrogen content (N); and (**d**) Carbon content (C). Significant correlations are denoted with an asterisk (***p < 0.001; **p < 0.01; *p < 0.05). (—Mean, □ Mean ± 1.96*SD, ⊥ Min-Max).

No differences were found in nitrogen or carbon isotope compositions (δ^15^N, δ^13^C) between *Quercus* species (Figure 1a,b, respectively), with δ^15^N ranging from −1.1‰ to 6.9‰ and δ^13^C from −27.3‰ and −20.0‰. Contrastingly, the nitrogen and carbon contents differed significantly among species (Figure 1c,d, respectively), with mean contents of 0.7% ± 0.1% (N) and 45.4% ± 0.5% (C) in QR acorns, and of 0.9% ± 0.2% (N) and 44.5% ± 0.9% (C) for QS acorns. To the best of our knowledge, scarce information regarding acorns’ nitrogen and carbon isotopic composition or N and C contents is available in bulk samples with values of ca. −21.0‰ for δ^13^C of *Q. ilex* being reported [19,20].

Considering the aridity range of our study and natural distribution of studied *Quercus* species—QR more interior (drier) than QS (coastal; more humid)—negative correlations between soil δ^15^N and AI and positive with soil C/N and soil N can be expected [31]. Delgado-Baquerizo et al. [32], while evaluating Mediterranean arid and semiarid grasslands, observed that aridity had the strongest relationship with N availability; they found a negative correlation between N and aridity, which suggests that increasing aridity (predictable under current climate change scenarios) would reduce N availability and consequently affect plant nutrient uptake and net primary production throughout this region. Moreover, Wang et al. [31], while evaluating N-cycling in arid and semi-arid grasslands, found that in areas with 0.32 < AI < 0.57 (similar to our gradient), the ecosystem N retention rate increased with increasing AI (thus, lower soil δ^15^N in less dry areas). In such gradient, N leaching losses, gaseous N losses (by soil nitrification and denitrification) and net plant N accumulation all contribute to N losses which can be related to the effects of rainfall on rates of N-cycling microbial activities. Then again, plant N uptake can increase as a result of higher water availability. In this study, non-significantly higher δ^15^N values and significantly lower N contents in QR acorn samples (from more arid sites, lower AI values) were found regarding QS samples (more coastal/humid sites, higher AI values). Therefore, it is possible that acorn δ^15^N signatures are likely reflecting soil N conditions and N-cycling (more N losses in drier sites). Nevertheless, in the studied area, N transformation rates might also be largely driven by variations in soil moisture rather than from the direct drivers of soil N availability. It is possible that the relationship between aridity and N availability could be driven by indirect effects operating over long-time scales (decades to millennia), including both biotic (e.g., plant cover) and abiotic (e.g., soil OC and pH). Considering acorn N patterns and if these factors are in fact more preponderant than short- and long-term direct effects of precipitation on N transformation rates, then we might expect to observe a lagged decrease in N availability in response to increasing aridity.

**Figure 2:**
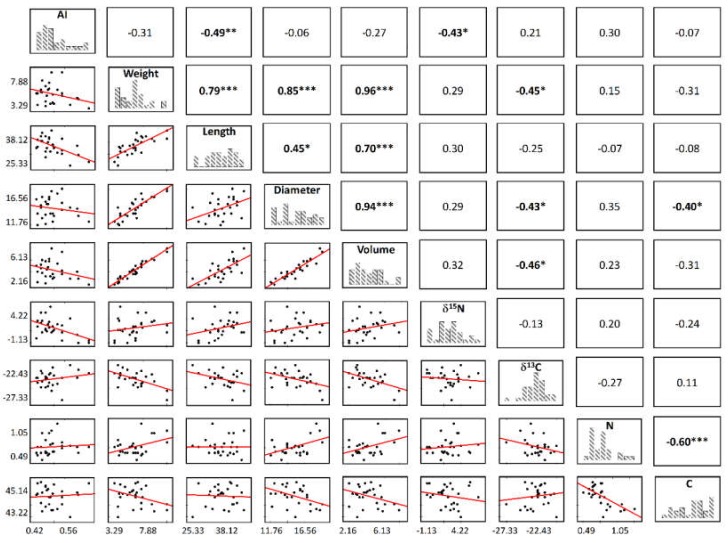
Correlations (Spearman rho) between acorns morphological and physiological traits and the aridity index (AI) (n = 28). Significant correlations are denoted with an asterisk in boldface (*** *p* < 0.001, ** *p* < 0.01, * *p* < 0.05).

In view of the non-significant differences (*p* > 0.05) between *Quercus* species regarding isotopic composition, the dataset was further analyzed disregarding the species effect. To test the association between morphological and physiological traits with the aridity index (AI), we used Spearman correlations (Figure 2).

With this approach, we found a negative relation between acorns’ δ^15^N and the aridity index (rho = −0.43, *p* < 0.05): a δ^15^N depletion is observed with increasing AI, meaning that more arid sites (lower AI) are conducive to a ^15^N enrichment in acorn samples. The sources of variation in N isotope natural abundance across ecosystems are still far from fully understood. Some studies report that increases in mean annual precipitation are conducive to increases in available N concentration [33,34], while others found the opposite [35,36] or report conflicting responses [37,38]. In addition, regarding temperature, inconsistent reports are found: some studies show that increasing temperature increases N availability [39,40], and others report no significant effects on soil N concentration [41]. The reported inconsistencies may result from differences in studied plant species, soil types, and ecosystems, which may determine the effects of temperature and precipitation on soil N availability. Nonetheless, our results are consistent with previous reports [31,37,42], which indicate that N-cycling in drier sites sustains more losses than in more humid ones. The negative association between AI and δ^15^N is possibly indicating higher losses in the lighter isotope while the soil is being enriched with the heavier one. In general, plant δ^15^N values are reduced with increasing annual precipitation (i.e., increasing AI), likely due to the mobilization of “lighter” N for plant nutrition [31]. It has been shown that, in a range of grasses and forbs, foliar δ^15^N reflects soil net nitrogen mineralization rates and the uptake ratio of NO_3_^−^ to NH_4_^+^ [43]. When water is freely available, mineral N release may occur and, consequently, delivers ^15^N depleted NO_3_, contributing to lower δ^15^N in plant tissues. Moreover, in a tropical context, Houlton et al. [44] demonstrated that the major determinant of N isotopic discrimination across a climate gradient was gaseous N loss by microbial denitrification. It was shown that N losses by denitrification were intensified by higher mean annual precipitation (MAP).

Still, acorns reflect aridity conditions, which might suggest long-term water limitation and probably soil nitrogen availability due to the observed ^15^N depletion along the AI gradient. Taking also into account the positive correlation between AI and acorns length (rho = −0.49, *p* < 0.01) (seed investment), these established relations support the use of acorns as a tool to access integrative effects of long-term annual climatic changes in response of trees to an aridity gradient. As for δ^13^C values, even though no correlation was found with AI (rho = 0.21), its negative relation (rho = −0.45; *p* < 0.05) with acorn weight hints that the greater is the water stress (more δ^13^C depleted samples) the higher is the plants’ investment in its seed size.

δ^13^C variation can be considered as indicators of differences in water availability, precipitation amount, temperature, and air relative humidity between the different geographical origins [45]. Usually, less negative δ^13^C values in more arid areas are found due to increasing drought and water resource shortages, a consequence of stomatal control in dry conditions. As can be observed (Figure 2), no significant correlation was found between δ^13^C and AI, which might suggest that acorns respond to other drivers such as local ones (e.g., plant cover, plant-soil interactions, plant phenology, local soil organic carbon, short-term precipitation or other climatic variables, and variability of water availability) rather than long-term climatic ones (e.g., MAP).

**Figure 3:**
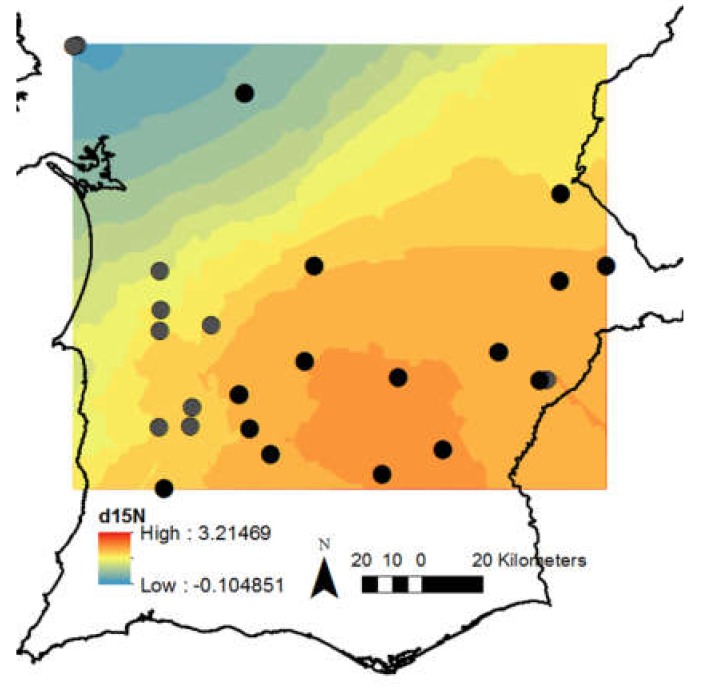
Acorn isoscapes of δ^15^N in the Alentejo region, Portugal.

All these relations can be perceived as a first approach towards determining the geographical origin of the black Iberian pig. Its intrinsic characteristics could, in fact, be framed into the ecosystem unique dynamics using acorn isotopes—the main source of feed. Thus, acorn isoscapes of nitrogen were developed as a stepping stone for the provenance of the Alentejano pig. Multivariate analysis and geospatial data analysis were applied to illustrate the result of the correlation between acorn δ^15^N isotopic composition and environmental variables, as shown in Figure 3.

**Figure 4:**
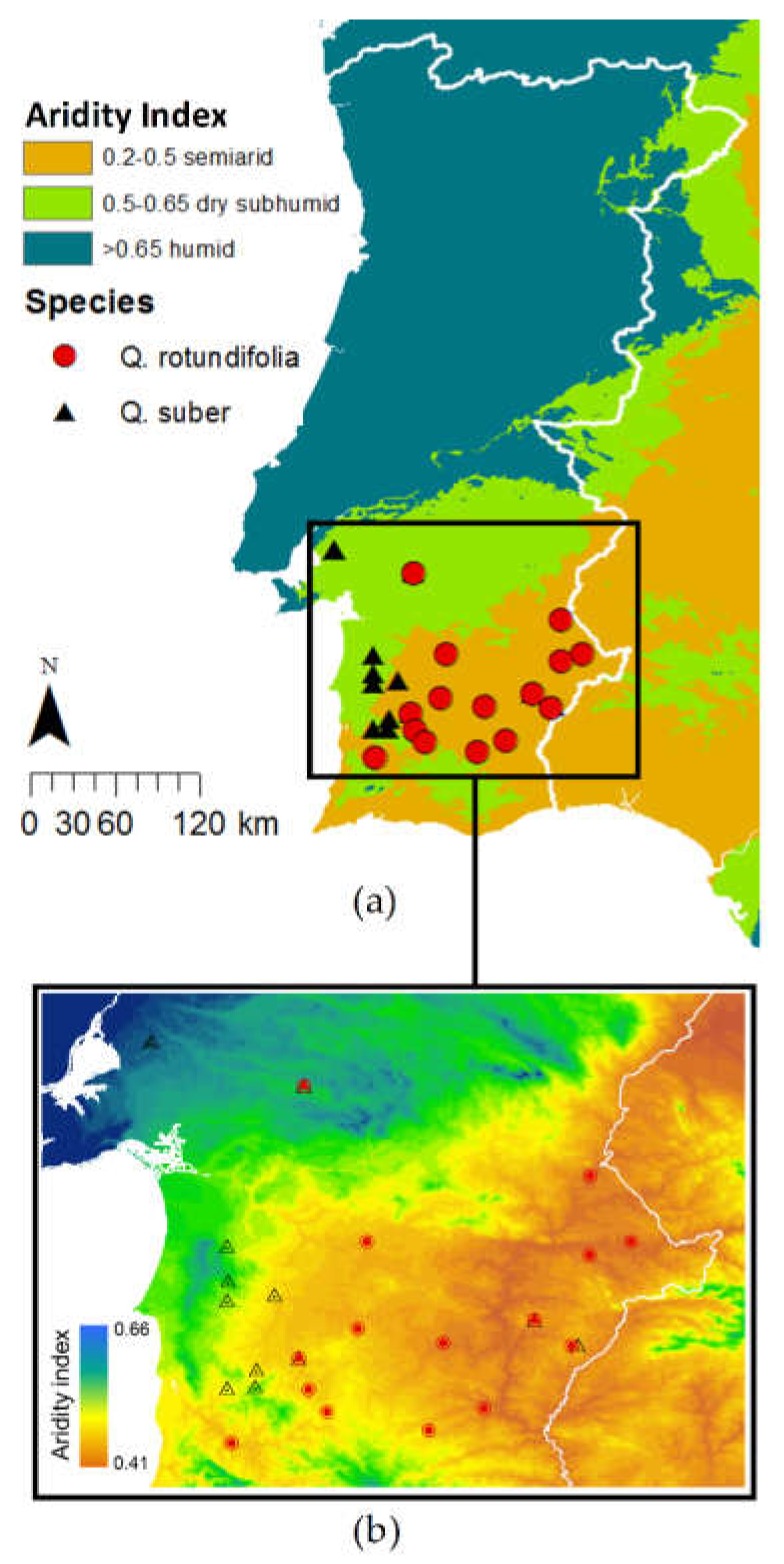
Study site: (**a**) sampling sites for *Quercus rotundifolia* (red) and *Q. suber* (black) within the aridity index gradient (lower values of the index indicate more arid areas); and (**b**) sampling sites along the aridity gradient, from low (blue) to high (orange) aridity. White line indicates the Portuguese border.

The influence of long-term climatic variables on acorn nitrogen signature is clearly demonstrated in its δ^15^N isoscapes (Figure 3). The δ^15^N values are distributed according to aridity index map (Figure 4b), where higher δ^15^N values are found in more dry areas. In contrast, no major trend was observed in acorns δ^13^C isoscapes (data not shown), leading to believe that this is a more site-specific response. The site-specific response for δ^13^C could be indicative that acorn’s isotopic traits respond to different local drivers. Nonetheless, the acorn δ^13^C site-specific signatures will potentially enable traceability for the Alentejano pig (correlations with pig fat δ^13^C signatures, currently under development by this research team).

## 3. Materials and Methods

### 3.1. Study Sites

The study was conducted in Mediterranean cork-oak, holm-oak, and mixed *Montados* randomly distributed at the Portuguese Alentejo region (Figure 4a), as to cover main production areas of the Alentejano pig breed (validated by the *Associação de Criadores de Porco Alentejano* (ACPA), association of Alentejano pig breeders) and to account with variability in climatic characteristics. Twenty-eight sampling sites were distributed in *Montados* throughout a spatial gradient of aridity: 15 for *Quercus rotundifolia* (QR) and 13 for *Quercus suber* (QS). The aridity index values (Figure 4b) ranged from 0.422 to 0.627 (data for the period of 1970–2000 obtained from the CGIAR-CSI global aridity database (http://www.cgiar-csi.org/data/global-aridity-and-pet-database) [46]). For QR sampling sites, the aridity index varied from 0.422 to 0.582, while in QS sites it ranged from 0.446 to 0.627. Five to ten adult oak trees (QR and QS) were selected in each sampling site. Ten acorns were sampled from random branches per individual tree between October and December 2017.

### 3.2. Morphological and Physiological Traits

The 28 samples of *Q. rotundifolia* and *Q. suber* were analyzed for acorn morphological parameters (weight, length, diameter, and calculated volume) and ecophysiological traits, the latter including acorn C and N concentrations and isotope ratios (δ^15^N and δ^13^C). Acorns (10 per sampling site) were weighted on an analytical scale (Startorius BP2215), length and diameter recorded (digital caliper; 0.05 mm resolution) and the volume (prolate spheroid) calculated according to the equation V = 4/3 × π × a^2^ × b, where *a* is the minor radius (diameter/2) and *b* is the major radius (length/2) of the acorns.

After main pericarp removal, the bulk acorn samples were dried at 60 °C for 48 h and milled to fine powder in a ball mill (Retsch MM 2000, Haan, Germany) for isotopic and elemental analysis. A homogenous composite sample was considered per site (total samples = 28). Stable isotope analysis of bulk acorn samples was carried out at the Stable Isotopes and Instrumental Analysis Facility (SIIAF, Centre for Ecology, Evolution and Environmental Changes, Faculty of Sciences, University of Lisbon, Portugal). Acorn δ^15^N and δ^13^C and C and N concentrations (%) were determined by continuous flow isotope ratio mass spectrometry (CF-IRMS) on a Sercon Hydra 20–22 (Sercon, UK) stable isotope ratio mass spectrometer, coupled to a EuroEA (EuroVector, Italy) elemental analyzer. The isotope ratios were expressed in δ‰ against Vienna-Pee Dee Belemnite for δ^13^C and air for δ^15^N according to δ‰ = [(R_sample_—R_reference_)/R_reference_] × 1000, where R is the ratio between the heavier isotope and the lighter one. The values were calculated against working in-house standards, calibrated against international reference materials: USGS25 and USGS35 (IAEA-International Atomic Energy Agency, Vienna, Austria; δ^15^N = −30.4 ± 0.4‰ VAIR and δ^15^N = +2.7 ± 0.2‰ VAIR, respectively) for δ^15^N; IAEA-CH-7 (IAEA (International Atomic Energy Agency), Vienna, Austria; δ^13^C = −32.151 ± 0.05‰ vPDB) and BCR-657 glucose (Community Bureau of Reference of the European Commission; δ^13^C = −10.76 ± 0.04‰ vPDB) for δ^13^C. Isotope ratio analysis uncertainty per batch was ≤ 0.1‰, calculated using 6–9 replicates of secondary isotopic reference material interspersed among samples.

### 3.3. Statistical Analysis

Statistical analysis was carried out using Statistica™v.8 Software (StatSoft, Tulsa, OK, USA; [47]). Differences between species (morphological and physiological variables) were determined by a non-parametric analysis of variance (Kruskal-Wallis test) as the assumption of normality was not found. Spearman correlation coefficients were also generated between the studied responses and environmental variables.

The aridity index (AI) was selected as the environmental variable as it reflects a measure of dryness, representing the ratio of mean annual precipitation to the mean annual potential evapotranspiration (long-term means of 1970–2000) [48] as the predictor for morpho- and physiological traits. AI data for the period 1970–2000 were retrieved from a global aridity database (http://www.cgiar-csi.org/data/global-aridity-and-pet-database) [46], and site-specific values extracted in ArcGIS^®^ (v10.5.1). The aridity index is strongly related to both mean annual precipitation (rho = 0.98, *p* < 0.001) and potential evapotranspiration (rho = −0.90, *p* < 0.001) in our study sites.

Mean acorn δ^13^C and δ^15^N, calculated from the average of 10 acorns from each site, was interpolated within the study area by ordinary kriging using Geostatistical Analyst Extension of ArcMap (ArcGis 10.5.1. for desktop). Experimental semi-variograms were fitted by a spherical isotropic function. Regarding δ^13^C, the measured sample points were not spatially autocorrelated, as the semivariogram did not depicts a fitted model, thus no robust mapping was achieved. Acorn δ^15^N samples showed spatial autocorrelation (semi-variogram fitted model: Nugget effect = 0.3 × 10^−1^; Sill = 0.45 × 10^−1^; Range = 0.94 distance degrees) and interpolation maps were produced in ArcGis 10.5.1.

## Figures and Tables

**Table 1:** Morphological traits of acorns from two *Quercus* species: *Q. rotundifolia* (n = 15) and *Q. suber* (n = 13).

Morphological Trait	*Quercus rotundifolia* (QR)	*Quercus suber* (QS)
Weight (g)	5.64 ± 1.21 (3.91–7.48)	6.18 ± 2.45 (3.29–10.18)
Length (mm)	37.57 ± 3.24 (33.39–43.50)	33.80 ± 5.88 (25.33–44.51)
Diameter (mm)	14.75 ± 1.59 (11.76–16.85)	15.49 ± 2.50 (11.93–18.96)
Volume (cm^3^)	4.34 ± 1.06 (2.58–6.03)	4.51 ± 2.05 (2.16–8.11)

Values are mean ± SD. Values between brackets correspond to the minimum and maximum value. Between species, no significant differences were found (Kruskal-Wallis test).

**Table 2:** Correlations (Spearman rho) between Aridity index (AI) and acorn weight, length, diameter, and volume according to *Quercus* species (*Q. rotundifolia* (QR) in light grey and *Q. suber* (QS) in dark grey).

		Weight	Length	Diameter	Volume	AI	
**Weight**		1.00	0.69 **	0.75 **	0.91 ***	−0.19	**QR**
**Length**	**QS**	0.93 ***	1.00	0.17	0.47	−0.17	
**Diameter**		0.90 ***	0.74*	1.00	0.94 ***	−0.08	
**Volume**		0.98 ***	0.91 ***	0.93 ***	1.00	−0.14	
**AI**		−0.57 *	−0.62 *	−0.32	−0.54	1.00	

Significant correlations are denoted with an asterisk (*** *p* < 0.001, ** *p* < 0.01, * *p* < 0.05). n = 15 for *Q. rotundifolia* (QR) and n = 13 for *Q. suber* (QS).
